# Photobiomodulation and Oxidative Stress: 980 nm Diode Laser Light Regulates Mitochondrial Activity and Reactive Oxygen Species Production

**DOI:** 10.1155/2021/6626286

**Published:** 2021-03-03

**Authors:** Andrea Amaroli, Claudio Pasquale, Angelina Zekiy, Anatoliy Utyuzh, Stefano Benedicenti, Antonio Signore, Silvia Ravera

**Affiliations:** ^1^Faculty of Dentistry, Department of Orthopaedic Dentistry, First Moscow State Medical University (Sechenov University), 119991 Moscow, Russia; ^2^Laser Therapy Centre, Department of Surgical and Diagnostic Sciences, University of Genoa, 16132 Genoa, Italy; ^3^Faculty of Dentistry, Department of Therapeutic Dentistry, First Moscow State Medical University (Sechenov University), 119991 Moscow, Russia; ^4^Department of Experimental Medicine, University of Genoa, 16132 Genoa, Italy

## Abstract

Photobiomodulation with 808 nm laser light electively stimulates Complexes III and IV of the mitochondrial respiratory chain, while Complexes I and II are not affected. At the wavelength of 1064 nm, Complexes I, III, and IV are excited, while Complex II and some mitochondrial matrix enzymes seem to be not receptive to photons at that wavelength. Complex IV was also activated by 633 nm. The mechanism of action of wavelengths in the range 900–1000 nm on mitochondria is less understood or not described. Oxidative stress from reactive oxygen species (ROS) generated by mitochondrial activity is an inescapable consequence of aerobic metabolism. The antioxidant enzyme system for ROS scavenging can keep them under control. However, alterations in mitochondrial activity can cause an increment of ROS production. ROS and ATP can play a role in cell death, cell proliferation, and cell cycle arrest. In our work, bovine liver isolated mitochondria were irradiated for 60 sec, in continuous wave mode with 980 nm and powers from 0.1 to 1.4 W (0.1 W increment at every step) to generate energies from 6 to 84 J, fluences from 7.7 to 107.7 J/cm^2^, power densities from 0.13 to 1.79 W/cm^2^, and spot size 0.78 cm^2^. The control was equal to 0 W. The activity of the mitochondria's complexes, Krebs cycle enzymes, ATP production, oxygen consumption, generation of ROS, and oxidative stress were detected. Lower powers (0.1–0.2 W) showed an inhibitory effect; those that were intermediate (0.3–0.7 W) did not display an effect, and the higher powers (0.8–1.1 W) induced an increment of ATP synthesis. Increasing the power (1.2–1.4 W) recovered the ATP production to the control level. The interaction occurred on Complexes III and IV, as well as ATP production and oxygen consumption. Results showed that 0.1 W uncoupled the respiratory chain and induced higher oxidative stress and drastic inhibition of ATP production. Conversely, 0.8 W kept mitochondria coupled and induced an increase of ATP production by increments of Complex III and IV activities. An augmentation of oxidative stress was also observed, probably as a consequence of the increased oxygen consumption and mitochondrial isolation experimental conditions. No effect was observed using 0.5 W, and no effect was observed on the enzymes of the Krebs cycle.

## 1. Introduction

Oxidative stress from reactive oxygen species (ROS) generated by mitochondrial activity is an inescapable consequence of aerobic metabolism. The vital role of mitochondria in tissue energy metabolism is to convert the products of biotransformation to CO_2_ and water. For this to happen, enzymes of the electron transport chain (ETC), such as NADH-dehydrogenase (Complex I), succinate dehydrogenase (Complex II), cytochrome bc1 (Complex III), and cytochrome c oxidase (Complex IV), which pump protons from the matrix to the intermembrane space, are necessary, determining a proton gradient and the synthesis of adenosine triphosphate (ATP) by the enzyme F_o_-F_1_ ATP synthase (Complex V) [1]. When the ETC becomes saturated with electrons, it can pass to O_2_ by Complexes I and III and generate superoxide anions [1]. The generation of superoxide anions can also be correlated to the univalent reduction of oxygen by mitochondrial semiquinones [2]. In this way, more than 90% of ATP produced and 85–90% of oxygen breathed by aerobic cellular tissues are derived from mitochondria [2, 3], and under physiological conditions, between 0.2 and 2% of this activity is converted into ROS [4]. Mitochondria are also equipped with an antioxidant enzymatic system (AES) for ROS scavenging, which keeps them under control without causing damage to the cell [5]. However, alterations in the transfer of electrons and/or AES can cause electrons to accumulate on the ETC complexes and enhance ROS production. From a mitochondrial point of view, ROS and ATP can regulate cell homeostasis and play a role in cell death, cell proliferation, and cell cycle arrest.

Karu and Afanasyeva and Passarella and Karu first described the ability of red and near-infrared (NIR) light to interact with mitochondria [6, 7]. Indeed, light-cell interactions in nonplant cells, such as nonphotosynthesising prokaryotic and protozoan cells and animal cells, have been described [8]. Basically, when a photon interacts with a specific photoacceptor, its energy is absorbed to generate high-energy electrons. The excited molecule can lose its energetic status in the form of heat or fluorescence emission, or the absorbed light energy can be transferred to a photosystem molecule as an excited electron or state. In this way, the photosystem converts the photon's energy into chemical energy, thanks to the tricky process of electron transport and a proton gradient, ending with the conversion of ADP to ATP [9]. In plants, this process occurs in the chloroplast, whereas it occurs in photoacceptors in bacteria, and the conversion of ADP takes place in the inner part of the cell membrane. In other eukaryotic cells, electron transport occurs in the mitochondrial respiratory chain [7, 8]. Complex IV was shown to be activated *in vitro* by a red laser (632.8 nm, 15 mW, CW, dose 6.3 × 10^3^ J/m^2^, 10 s) [10]. Particularly, according to metal-ligand systems and absorption spectra, such as 450, 620–680, and 760–895 nm, characteristically different peaks may be related to oxidized or reduced copper into cytochrome c oxidase [11]. In previous papers, we showed that 808 nm electively stimulates Complex IV and that Complex III was excited poorly, while Complexes I and II were not affected [12]. In addition, by increasing the wavelength to 1064 nm, the photon and mitochondrial complex interaction changes, and Complexes I, III, and IV are affected, while the extrinsic mitochondrial membrane Complex II and mitochondrial matrix enzymes seem to be not receptive to photons at this wavelength [13].

The mechanism of action of wavelengths 900–1000 nm is, however, less well understood, and, in particular, interactions with mitochondrial complexes have not been described. Conversely, Wang et al. [14] concluded that at the parameters tested, 980 nm affected temperature-gated calcium ion channels but not mitochondrial cytochrome c oxidase when compared to 810 nm. Water could then be a candidate as a chromophore for longer wavelengths of NIR, based on its absorption spectrum.

Starting with these premises, the purpose of this study was to evaluate the interaction between a 980 nm diode laser light and mitochondrial activity. The investigators hypothesized that according to previous *in vitro* literature on 1064 nm wavelength, the 980 nm wavelength could have a modulatory effect on respiratory chain activities. The specific aims of the study were to determine the effectiveness of a 980 nm diode laser, irradiated at the powers from 0 to 1.4 W (0.1 W increment at every step), for 60 sec in continuous wave mode (CW), spot size 0.78 cm*^2^*, on bovine liver isolated mitochondria. The activity of the mitochondria's complexes, ATP production, oxygen consumption, and production of ROS were measured. The results were discussed and compared to our previous data and literature.

## 2. Materials and Methods

### 2.1. Laser Features, Parameters, and Method of Irradiation

The experimental design is shown in supplementary Figure 1. Mitochondria were isolated from bovine liver and irradiated as described, at room-air temperature, or partially immersed in water to prevent macroscopic thermal effects.

The laser device utilised in the study was the Wiser wireless diode laser by Doctor Smile–LAMBDA Spa (Vicenza, Italy). The 980 nm diode laser light was irradiated by the AB 2799 hand-piece (Doctor Smile-LAMBDA Spa, Vicenza, Italy). The AB 2799 hand-piece is a novel hand-piece with a flat-top beam profile, which was set following the manufacturer's specifications, to allow delivery of homogenous irradiation over the surface area with the same irradiation spot area (0.78 cm^2^) and power from contact, extending to many centimetres (~100 cm) of distance from the target; by comparison, the standard hand-piece would deliver a Gaussian profile of irradiation and is accompanied by beam divergence over distance [12, 15]. To make sure that the laser delivery power was constant during the irradiation mode and suitable for our experimental setup, the PM160T-HP power meter (ThorLabs, Germany) was utilised according to Hanna et al. [15].

To control the thermal increase on the irradiated samples, a thermal camera FLIR ONE Pro-iOS (FLIR Systems, Inc. designs, Portland, USA.) (dynamic range: -20°C/+400°C; resolution 0.1°C) was used during irradiation. The temperature measures were collected before and after irradiation, which was performed at room-air temperature (25°C) or with a tube sample partially immersed in 300 ml of water (25°C) (supplementary Figure 1). The temperature of the sample of mitochondria irradiated at the room-air temperature was also measured after the addition of reagents for biochemical evaluation (temperature of the reagents 25°C).

For our experimental purpose, the Wiser wireless diode laser was set to irradiate for 60 sec in continuous wave mode with power from 0.1 to 1.4 W (0.1 W increment at every step), which generated energies from 6 to 84 J, fluences from 7.7 to 107.7 J/cm^2^, and power densities from 0.13 to 1.79 W/cm^2^ (please see Table 1 for a more descriptive representation of the parameters).

Irradiations were performed with both the sample and the hand-piece fixed to a stand at a distance of 3.5 mm. Irradiations performed with the laser device kept off were considered a control.

### 2.2. Reagents

Salt, substrates, and all other chemicals (of analytical grade) were purchased from Sigma–Aldrich (St. Louis, MO, USA). Protein Molecular Weight (MW) markers were from Bio-Rad (Hercules, CA, USA). Ultrapure water (Milli-Q; Millipore, Billerica, MA, USA) was used throughout. Safety precautions were taken for chemical hazards in carrying out the experiments. Ampicillin (25 *μ*g/ml) was used in all the solutions, and sterile experimental conditions were employed where appropriate.

### 2.3. Mitochondrial Enriched-Fraction Isolation

Bovine liver from 2 female and 2 male cattle of less than 1 year of age were acquired from the slaughterhouse of Ceva, Torino, Italy. The cattle were bred for human consumption, following the directives of the Italian Ministry of Agricultural, Food and Forestry Policies. Samples were taken and processed immediately after slaughter, following all safety rules. Since the animals were not bred nor sacrificed at the University of Genoa, it was not necessary to request any ethical committee approval.

To obtain a mitochondrial enriched fraction, the bovine liver was washed in PBS and homogenized in a buffer containing 0.25 M sucrose, 0.15 M KCl, 10 mM Tris-HCl pH 7.4, and 1 mM EDTA. The homogenate was centrifuged at 800 × g for 10 min. The supernatant was filtered and centrifuged at 12000 × g for 15 min. Pellet was resuspended in another buffer containing 0.25 M sucrose, 75 mM mannitol, 10 mM Tris-HCl pH 7.4, and 1 mM EDTA. The final supernatant was centrifuged at 12000 × g for 15 min, and the mitochondrial pellet resuspended in the same buffer [16].

### 2.4. Oxygen Consumption Measurements

The oxygen consumption rate (OCR) was assayed in a thermostatically controlled oxygraph apparatus equipped with an amperometric electrode (Unisense–Microrespiration, Unisense A/S, Denmark) on mitochondrial enriched fraction treated or not with the 980 nm laser. For each sample, 50 *μ*g of total protein was used. The samples were incubated in the respiration buffer composed of 120 mM KCl, 2 mM MgCl_2_, 1 mM KH_2_PO_4_, 50 mM Tris-HCl pH 7.4, and 25 *μ*g/ml ampicillin. As respiratory substrates, 5 mM pyruvate+2.5 mM malate were added to the respiration buffer [12].

### 2.5. Evaluation of Mitochondrial ATP Synthesis

To evaluate the ATP production through the F_o_-F_1_ ATP synthase (ATP synthase), mitochondrial enriched fraction treated or not with the 980 nm laser was dissolved in a solution containing 100 mM Tris-HCl pH 7.4, 100 mM KCl, 1 mM EGTA, 2.5 mM EDTA, 5 mM MgCl_2_, 0.2 mM di(adenosine-5′) penta-phosphate, 0.6 mM ouabain, ampicillin (25 *μ*g/ml), 5 mM KH_2_PO_4_, and 5 mM pyruvate+2.5 mM malate, used as respiratory substrates. ATP synthesis started after the addition of 0.1 mM ADP and was monitored for 2 minutes, in a luminometer (Glomax 20/20, Promega) by the luciferin/luciferase chemiluminescent method. An ATP standard solution between 10^−9^ and 10^−7^ M was used for calibration [12].

### 2.6. Calculation of Mitochondrial Efficiency

To evaluate the energy production efficiency by the mitochondrial enriched-fraction, the ratio between the produced ATP and consumed atomic oxygen (P/O) was calculated. When the oxygen consumption is perfectly associated to the ATP synthesis, the P/O ratio is about 2.5 in the presence of pyruvate+malate as respiratory substrates [17]. Conversely, in the uncoupled status, this value decreases correlating to the grade of the oxidative phosphorylation inefficiency.

### 2.7. Assay of Respiratory Complex Activity

The activity of the four respiratory complexes was assayed on 50 *μ*g of total mitochondrial enriched-fraction protein treated or not with the 980 nm laser [13].

Complex I (NADH-ubiquinone oxidoreductase) was assayed following the reduction of ferricyanide at 420 nm; the assay solution was composed by 100 mM Tris-HCl pH 7.4, 0.6 mM NADH, 0.8 mM ferricyanide, 50 mM KCl, 5 mM MgCl_2_, 1 mM EGTA, and 50 *μ*M antimycin A.

Complex II (succinic dehydrogenase) activity was measured following the reduction of ferricyanide at 420 nm; the assay solution was composed by 100 mM Tris-HCl pH 7.4, 20 mM succinate, 0.8 mM ferricyanide, 50 mM KCl, 5 mM MgCl_2_, 1 mM EGTA, 20 *μ*M rotenone, and 50 *μ*M antimycin A.

Complex III (cytochrome c reductase) activity was assayed following the reduction of oxidized cytochrome c (Cyt c) at 550 nm; the assay solution was composed by 100 mM Tris-HCl pH 7.4 and 0.03% oxidized cytochrome c. The assay started with the addition of 0.7 mM NADH.

Complex IV (cytochrome c oxidase) was assayed following the oxidation of ascorbate-reduced Cyt c at 550 nm; the assay solution was composed by 100 mM Tris-HCl pH 7.4, 50 mM, and 0.03% reduced cytochrome c.

### 2.8. Isocitric Dehydrogenase and Malate Dehydrogenase Assay

Isocitric dehydrogenase (IDH; EC: 1.1.1.41) and malate dehydrogenase (MDH; EC 1.1.1.37) have been assayed as Krebs cycle markers. For each assay, 50 *μ*g of total mitochondrial enriched-fraction protein treated or not with the 980 nm laser was used.

For IDH, the assay solution contained 100 mM Imidazole buffer pH 8, 3.5 mM MgCl_2_, 0.41 mM NADP, and 0.55 mM isocitric acid [18].

For MDH, the assay solution is formed by 100 mM Tris-HCl pH 7.5, 0.5 mM oxaloacetic acid, and 0.2 mM NADH [13].

### 2.9. Superoxide Anion Assay

The production superoxide anion was assayed as the difference between total and SOD-inhibitable cytochrome c reduction. 50 *μ*g of total mitochondrial enriched-fraction protein treated or not with the 980 nm laser was added to 100 *μ*M cytochrome c and 300 U SOD, if present, and the changes in absorbance of reduced cytochrome c was measured spectrophotometrically, at 550 nm [19].

### 2.10. Evaluation of Lipid Peroxidation

To assess lipid peroxidation in mitochondrial enriched-fraction protein treated or not with the 980 nm laser, the malondialdehyde (MDA) level was evaluated, using the thiobarbituric acid reactive substances (TBARS). The TBARS solution contained 15% trichloroacetic acid (TCA) in 0.25 N HCl and 26 mM 2-thiobarbituric acid. To evaluate the basal concentration of MDA, 600 *μ*l of TBARS solution was added to 50 *μ*g of total protein dissolved in 300 *μ*l of Milli-Q water, after 10 minutes of laser exposure. The mix will be incubated for 40 min at 100°C, then centrifuged at 14000 rpm for 2 min, and the supernatant was analysed spectrophotometrically, at 532 nm [20].

### 2.11. Statistical Analysis

Statistical analyses were performed with GraphPad Prism software version 7 (GraphPad Software). All parameters were tested by one-way ANOVA followed by Bonferroni test. Data are expressed as mean ± standard deviation (SD) from 3 to 5 independent determinations performed in duplicate. In the figures, SD is shown as error bars. An error probability with *p* < 0.05 was selected as significant.

## 3. Results

### 3.1. Temperature Evaluation and Its Effect on the Results

As shown in supplementary Figure 2, when irradiated at the room-air temperature (blue line), the temperature of the irradiated mitochondrial samples progressively increased (from 25°C to 38°C) by increasing the power and energy irradiated (yellow line). However, when the reagents for the biochemical analysis were added, the final temperature recovered to the initial value of 25°C (green line). Conversely, the irradiation with the mitochondrial samples partially immersed in water allowed them to keep the temperature quite constant at 25°C (red line). Comparison of the results obtained after irradiation at the room-air temperature or in water did not show statistically significant differences.

### 3.2. 980 nm Laser Affects the OxPhos Activity

**Figure 1:**
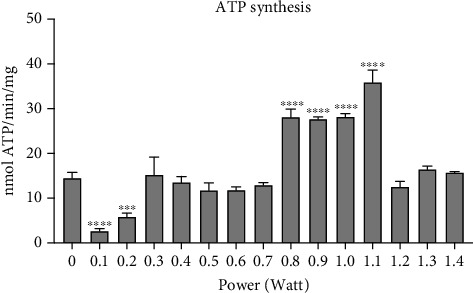
Effects of the 980 nm laser on F_o_-F_1_ ATP synthase activity. The graph shows the energy production by the F_o_-F_1_ ATP synthase in mitochondria irradiated with different powers (0-1.4 W) for 60 seconds; spot size 0.78 cm^2^. Each panel is representative of three independent experiments, and data are expressed as mean ± SD. ∗∗∗ and ∗∗∗∗ indicate a significant difference for *p* < 0.001 and 0.0001, respectively, between the nontreated sample (0 W) and the irradiated samples.

To evaluate whether the 980 nm laser exerted effects on the mitochondrial energy production, as already observed for the 1064 nm Nd:YAG laser [13], ATP synthesis through ATP synthase was evaluated applying a power of 0–1.4 W. As reported in Figure 1, the 980 nm laser induced a marked decrease in the ATP production when exposed to 0.1 and 0.2 W. Conversely, the power between 0.8 and 1.1 W determined an enhancement of energy synthesis, while the intermediate values (0.3–0.7 W) did not display effects. Based on these data, we chose to evaluate the energy metabolism of mitochondria exposed to the 980 nm laser, using the following power: 0, 0.1, 0.5, and 0.8 W.

**Figure 2:**
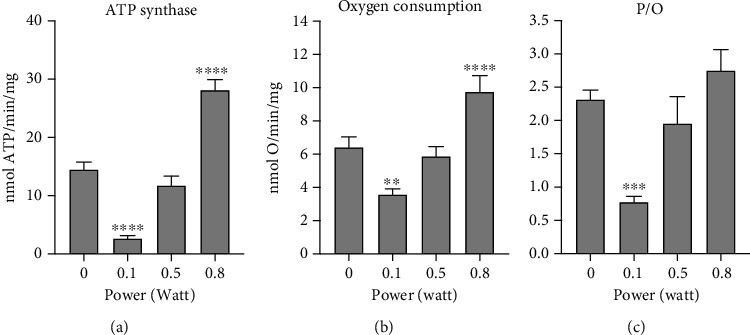
Effects of the 980 nm laser on mitochondrial OxPhos activity: (a) ATP synthase activity; (b) oxygen consumption rate; (c) P/O ratio, as a marker of the mitochondrial energy efficiency. Each panel is representative of three independent experiments, and data are expressed as mean ± SD. ∗∗, ∗∗∗, and ∗∗∗∗ indicate a significant difference for *p* < 0.01, 0.001, and 0.0001, respectively, between the nontreated sample (0 W) and the samples irradiated with different powers (0.1, 0.5, 0.8 W) for 60 seconds; spot size 0.78 cm^2^.

Data reported in Figure 2 show that the oxygen consumption rate (OCR) (panel b) followed the same trend observed for ATP synthesis (panel a) when mitochondria were stimulated with pyruvate and malate as respiratory substrates. Moreover, evaluating the efficiency of the mitochondrial energy production, we observed that the lower power determined not only a reduction of activity but also the decrement of the P/O ratio, which was around 0.7, instead of the regular value of 2.5 [17]. This suggested that 0.1 W induced an uncoupling status between the ATP production and respiration. In contrast, higher power did not affect mitochondrial efficiency (panel c).

### 3.3. Modulation of OxPhos Activity Induced by the 980 nm Laser Depended on Changes in the Activity of Complexes III and IV

**Figure 3:**
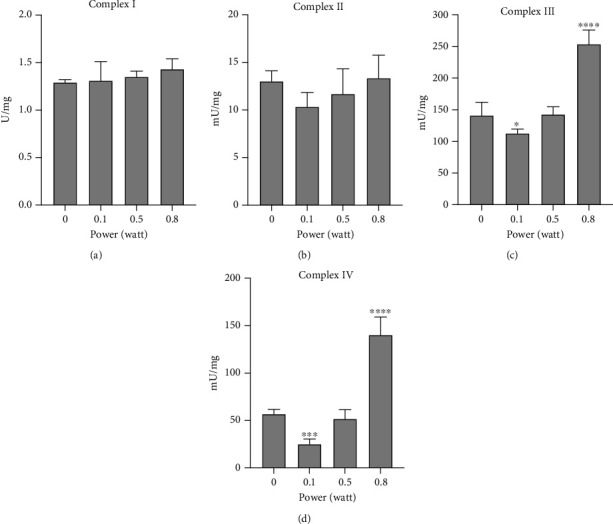
Effects of the 980 nm laser on respiratory complexes: (a) Complex I activity; (b) Complex II activity; (c) Complex III activity; (d) Complex IV activity. Each panel is representative of five independent experiments, and data are expressed as mean ± SD. ∗, ∗∗∗, and ∗∗∗∗ indicate a significant difference for *p* < 0.05, 0.001, and 0.0001, respectively, between the nontreated sample (0 W) and the samples irradiated with different powers (0.1, 0.5, 0.8 W) for 60 seconds; spot size 0.78 cm^2^.

To understand the targets of the 980 nm laser on the electron transport chain, the activities of the four respiratory complexes were evaluated. As reported in Figure 3, only Complexes III and IV were modulated by the laser (panels c and d), while Complexes I and II did not show a difference between untreated and treated samples (panels a and b). In particular, the lower power (0.1 W) determined a marked reduction of the activities of Complexes III and IV. Conversely, the higher power (0.8 W) induced an increment of the complexes' activity. No effects were observed on any complex with the 0.5 W treatment.

### 3.4. The 980 nm Laser Did Not Alter the Activity of Isocitric Dehydrogenase and Malate Dehydrogenase

**Figure 4:**
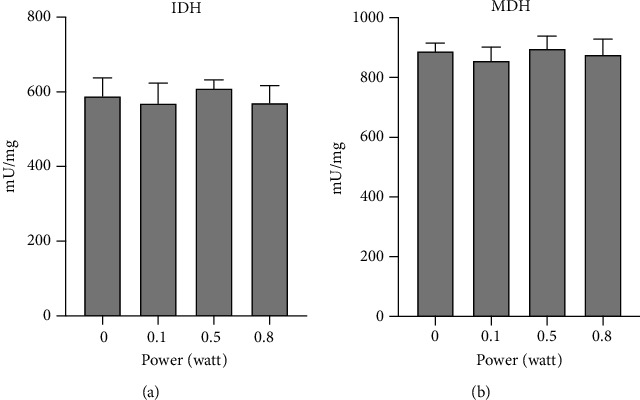
Effects of the 980 nm laser on isocitric dehydrogenase and malate dehydrogenase: (a) isocitric dehydrogenase activity (IDH); (b) malate dehydrogenase activity (MDH). Each panel is representative of three independent experiments, and data are expressed as mean ± SD. No significant difference has been observed between the nontreated sample (0 W) and the samples irradiated with different powers (0.1, 0.5, 0.8 W) for 60 seconds; spot size 0.78 cm^2^.

To evaluate whether the modulation of OxPhos activity could depend on the alteration of the Krebs cycles, the upstream pathway of the electron transport chain and activity of isocitric dehydrogenase (IDH) and malate dehydrogenase (MDH) were evaluated.  Figure 4 shows that the three considered powers (0.1, 0.5, and 0.8 W) did not exert effects on the activity of IDH (panel a) and MDH (panel b).

### 3.5. Modulation of OxPhos Activity by the 980 nm Laser Determined an Increment of Superoxide Production and Lipid Peroxidation

Mitochondria are considered one of the principal sources of oxidative stress production, which increases when the ATP production and oxygen consumption are uncoupled [21]. Therefore, to evaluate whether OxPhos modulation due to the laser treatment could be associated with an increment of the oxidative stress production and accumulation of oxidative damage, the superoxide level and the malondialdehyde concentration were evaluated.

**Figure 5:**
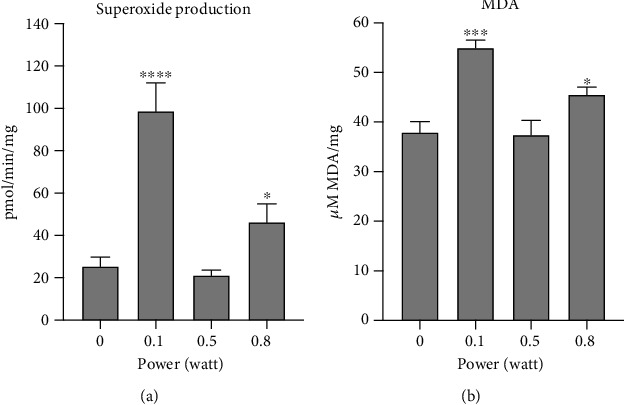
Effects of the 980 nm laser on oxidative stress production and lipid peroxidation: (a) superoxide production; (b) malondialdehyde (MDA) level. Each panel is representative of three independent experiments, and data are expressed as mean ± SD. ∗, ∗∗∗, and ∗∗∗∗ indicate a significant difference for *p* < 0.05, 0.001, and 0.001, respectively, between the nontreated sample (0 W) and the samples irradiated with different powers (0.1, 0.5, 0.8 W) for 60 seconds; spot size 0.78 cm^2^.

Figure 5(a) shows that both 0.1 and 0.8 W determined an increment of the superoxide production, although more evident was the effect of the lower power. In contrast, the increment observed after the treatment with 0.8 W may be associated with the increment of OxPhos enhancement.

The same trend was observed by evaluating the MDA intracellular concentration (Figure 5(b)), as a marker of lipid peroxidation, suggesting that the increment of oxidative stress was associated with oxidative damage of the mitochondrial membrane. No oxidative stress and lipid peroxidation were observed when the sample was treated with 0.5 W.

## 4. Discussion

The interaction between light and cells is well-known in vegetable cells. Red and NIR light is, however, able to modulate nonplant cell energetic metabolism, and this happens because of a parallel and convergent evolution of both chloroplasts and mitochondria from ancestral bacteria [8], known as the theory of endosymbiont models. The relative medical subject is known as photobiomodulation and can lead to improvement of pathological conditions [22]. Our data describe for the first time the ability of 980 nm light to interact with the respiratory chain of bovine liver mitochondria. Under our experimental conditions, a diode laser light was used to irradiate cells for 60 seconds, and three different effects were observed on ATP production. Lower powers (0.1–0.2 W) showed a sizable, inhibitory effect; intermediate powers (0.3–0.7 W) had no effect; and the higher powers (0.8–1.1 W) induced a strong and stable increment of ATP synthesis, which reached a peak at 1.1 W. Increasing the power (1.2–1.4 W) recovered the ATP production to the control level.

Because of the isolated experimental conditions of the mitochondria, we do not know what the consequences of cell homeostasis would be. However, the data suggest a reconsideration of the parameters able to interact with cell photoacceptors. Essentially, the statement that higher energies and powers have undoubtedly damaging effects with respect to the lower, which are “curative” [23, 24], should be reassessed. Actually, our data pointed out that a hormetic behaviour can certainly occur, but into narrow windows of positive effect/no effect/negative effect more than a watershed upper at or lower of. Additionally, the effectiveness of 980 nm joined to previous works resulted in a laser light and mitochondrial interaction of red and NIR wavelengths [7, 12, 13, 25], pointing to photobiomodulation on a wider spectrum of wavelengths than photosynthesis. Indeed, all life forms need energy for existence. However, only photosynthetic organisms developed light-energy conversion, and by evolution, ranges of light intensities have been selected for better survival in the biosphere [26]. The animal cell did not choose sunlight as a source of energy for its metabolism, and most cell types have no interaction with light; from this point of view, only cells interacting with light evolved the ability to use specific light stimuli, for instance, for vitamin D production and the vision. Interaction between mitochondria and 980 nm light prevalently occurs with Complex IV, as shown in our results, despite the fact that Complex III activity is also deeply stimulated. Complexes I and II were not energized. Because of similar experimental conditions with bovine isolated mitochondria from liver tissue, the data showed here and our previous works on mitochondrial complexes can be compared.

Complex IV was similarly stimulated moving from 808 to 980 and 1064 nm. Complex III was also excited by the same wavelengths, but the intensity progressively increased when compared to the other complexes. Complex I was conversely stimulated only by 1064 nm, while Complex II never changed. Besides, Complex IV was shown to be activated *in vitro* by 633 nm [10].

The respiratory chain is situated in the inner membrane of mitochondria and comprises four multimeric protein complexes: Complex I containing eight Fe-S clusters implicated the electrons' transfer from reduced flavin mononucleotide (FMNH2) to ubiquinone; Complex II has a heme b prosthetic group in its anchor domain, essential for the structural integrity and its function; Complex III contains cytochrome b subunit and two heme moieties, a cytochrome c1 subunit with one heme, and a Rieske protein subunit (UQCRFS1) with a (2Fe-2S) cluster; and Complex IV that modulates the final step in the ETC, by catalysing the reduction of O_2_ to water. It presents two heme moieties and two Cu centres, participating in the electron transfer process [27].

As previously discussed in our paper on the characterization between 1064 nm light and mitochondrial complexes, explaining the change as a mundane variation of light–“metal” uptake is an implausible choice. This model was not able to explain the unaffected behaviour of Complex I and, in the second analysis, since both iron and copper coefficients of absorption did not significantly increase or change, respectively, increasing wavelength up to 1064 nm [28, 29].

Conversely, water had a weak absorption in the visible spectrum but it increased by moving toward the 1064 nm. Additionally, despite lipids in the near-infrared wavelength region, there were two high peaks at 1210 and 1720 nm and a third lesser peak in the range of 900–1000 nm [30]. Thus, the light-water interaction at the nanoscale level could modify cell membrane features and the activities of the intramembrane complexes. In contrast, OxPhos machinery function depends on the integrity of the inner mitochondrial membrane, since the protons transported by the respiratory complexes should not be free to pass the membrane. Protons must be returned to the mitochondrial matrix only through the F_o_ moiety of the ATP synthase, to produce energy efficiently [31, 32]. Thus, the alteration of lipids constituting the inner mitochondrial membrane could cause a change in its shape and energy metabolism [33]. In particular, the laser could affect the structure of cardiolipin, a phospholipid expressed only in the active respiring membranes [34], playing a pivotal role in the structure of the respiratory complexes [35] and proton transport [36].

However, it is important to note that respiratory Complexes I, III, and IV, but not Complex II, are embedded in the inner mitochondrial membrane, as well as the F_o_ moiety of ATP synthase. This suggests that Complex II could be less prone to be affected by laser treatment. Moreover, it has been demonstrated that only Complexes I, III, and IV, as well as ATP synthase, are organized in a super complex, probably to increase electron transport and make the relative energy production more efficient [37–39]. However, the irradiation could interact not only with the cytochromes that constitute the respiratory complexes but also with ubiquinone or cytochrome c, the electron shuttles between Complexes I or II with III, and between III and IV, respectively.

Contextually, Pasternak et al. [40] demonstrated that 808 and 905 nm laser light may induce functional and structural modifications in cell membranes.

The H_3_O^+^ and OH^−^ ions are important species in biology, chemical physics, and electrochemistry, which carry acid-base reactions and the charge transfer process in aqueous solutions, by serving as intermediates for protonic transport [41]. Recently, authors studied the infrared spectra of pure water and mixtures to show the hidden IR vibrations of water's ionic species; IR spectrum affected it and was partially defined by the dynamics of ionic species, which were influenced. Interestingly, Walski et al. [42] suggested the polychromatic light (750–2000 nm) disturbs the energy of hydrogen bonds, increases water molecules dissociation, and consequently, alters biological membrane features. In particular, these alterations could affect particular lipids, such as cardiolipin mentioned above, which are involved in the ability of electron transport between the respiratory complexes and/or in the proton transport through them, altering OxPhos functionality.

Indeed, the paradigm of mechanical vibrations of molecules is one of the accredited models that explain nonionizing radiation effects on cells, by interaction with water, transmembrane proteins, and phospholipidic bilayers to produce changes in membrane fluidity, permeability, and protein activities [43–45].

As mentioned in the introduction, ROS are products of oxygen metabolism, as the natural consequence of mitochondrial respiration in all aerobic organisms. However, under physiological conditions, mitochondria keep ROS levels under control by a system of ROS-scavenging that prevents reactive species increment and, thus, cell damage [5]. In this way, mitochondria and low concentrations of ROS can play a pivotal role in cellular homeostasis through modulation of cellular signalling pathways.

Conversely, when there are high levels of ROS and the production/scavenging system becomes unbalanced, oxidative stress occurs [2], leading to indiscriminate damage to biological molecules and loss of cell functions and cell death.

Our data showed some irradiation can induce increments of ROS production. However, we must make a distinction between the lower and higher dose effect, in terms of the “amount of” and “modality with.” In particular, the increment of superoxide production observed after irradiation with 0.1 W probably depends on the uncoupled status, although the OxPhos activity was reduced in comparison to the control. This depends on the fact that mitochondria are considered one of the principal sources of oxidative stress production, which increases when ATP production and oxygen consumption are uncoupled [21]. Moreover, increased oxidative stress production could induce a vicious circle in which the alteration of OxPhos activity due to the laser treatment increases the oxidative stress that, in turn, negatively affects OxPhos activity [46]. This hypothesis was confirmed by the massive accumulation of MDA, which suggested that oxidative stress was not counterbalanced by sufficient antioxidant defences, despite mitochondria expressing both catalase and superoxide dismutase type 2 as antioxidant enzymes. In contrast, we worked with isolated mitochondria, which may have partly lost detoxifying enzymes and scavenger molecules during their preparation. Moreover, the monoelectron transport between the respiratory complexes determines an easier oxidative stress production, both in terms of reactive oxygen species and lipid peroxidation, whether the respiratory complexes and the relative shuttle are altered.

Conversely, the increment of superoxide production and MDA accumulation in mitochondria irradiated with 0.8 W could be associated simply with the increment of OxPhos activity, which, in perfect coupled conditions, produced oxidative stress from Complexes I and III [47, 48].

Chinopoulos and Adam-Vizi [49] described that in addition to the mitochondrial respiratory chain, the Krebs cycle should also have a role in mitochondrial ROS management. However, our data showed no effect of 980 nm laser light on IDH and MDH, as well as Complex II (called also succinic dehydrogenase, one of the eight enzymes involved in the Krebs cycle other than the second respiratory complex). This suggested that in our experimental conditions, ROS formation was due to the modulation of respiratory chain activity that occurs as a consequence of light-photoacceptor interaction (copper, iron, water) more than a generic increment of temperature, increments of temperature that were macroscopically considered, measured, and avoided in our experimental design.

Notably, oxidative stress could be induced in the cell by light-mitochondrial interaction through an alternative way than the respiratory chain and oxygen consumed. In this respect, in our previous works, we demonstrated that “808 nm and 980 nm infrared laser light directly affect the stored Ca^2+^ homeostasis, independent of the mitochondrial respiratory chain activities” [50]; mitochondria are one of the major reserves of sequestered calcium [51]. Additionally, we also showed a connection between the modulation of calcium homeostasis and both nitric oxide production and glutamate release in a unicellular organism [52] and the murine nerve terminal [53]. The reciprocal relationship among ROS formation elevated intracellular calcium concentration, and the/mitochondrial death is known [49]. Therefore, PBM besides both positive stimulation and support of cell metabolism could, if wrongly administered, affect mediators of cell injury, such as Ca^2+^, ROS, and reactive nitrogen species formation and latent glutamate-induced delayed calcium deregulation [8].

## 5. Conclusions

In conclusion, we showed for the first time the ability of the 980 nm diode laser light to interact with the mitochondria from bovine liver. The interaction behaved as a window effect and interested Complexes III and IV, as well as ATP production and oxygen consumption. Investigation of the effect of 0.1 W power irradiated for 60 sec highlighted that photobiomodulation can uncouple the respiratory chain, induce higher oxidative stress, and cause drastic inhibition of ATP production. Conversely, 0.8 W kept mitochondria coupled and induced increments of ATP production by increments of Complex III and IV activities; an increment of oxidative stress was also observed, as a likely consequence of the increased oxygen consumption and mitochondrial isolation experimental conditions.

## Figures and Tables

**Table 1:** Parameters irradiated by the 980 nm wiser wireless diode laser.

Power (W)	0.10	0.20	0.30	0.40	0.50	0.60	0.70	0.80	0.90	1.00	1.10	1.20	1.30	1.40
Continuous-wave mode	Yes	Yes	Yes	Yes	Yes	Yes	Yes	Yes	Yes	Yes	Yes	Yes	Yes	Yes
Irradiation time (sec)	60.00	60.00	60.00	60.00	60.00	60.00	60.00	60.00	60.00	60.00	60.00	60.00	60.00	60.00
Spot area (cm^2^)	0.78	0.78	0.78	0.78	0.78	0.78	0.78	0.78	0.78	0.78	0.78	0.78	0.78	0.78
Energy (J)	6.00	12.00	18.00	24.00	30.00	36.00	42.00	48.00	54.00	60.00	66.00	72.00	78.00	84.00
Fluence (J/cm^2^)	7.69	15.38	23.08	30.77	38.46	46.15	53.85	61.54	69.23	76.92	84.62	92.31	100.00	107.69
Power density (W/cm^2^)	0.13	0.26	0.38	0.51	0.64	0.77	0.90	1.03	1.15	1.28	1.41	1.54	1.67	1.79

## Data Availability

The data used to support the findings of this study are available from the corresponding author upon request.

## Supplementary Materials

Supplementary Materials Figure 1 supplementary: experimental design. Mitochondria were isolated from bovine liver. Mitochondria samples were irradiated at the room-air temperature or with the tube sample immersed in water. The samples were then processed for the biochemical analysis. Figure 2 supplementary: representations of sample temperature behaviour during the experiments. Before: temperature of the sample before irradiation. Room-air after: temperature of the sample after irradiation performed at room-air temperature. Room-air after+reagents: temperature of the sample after irradiation performed at a room-air temperature and the addition of reagents for biochemical evaluation. Water after: temperature of the sample after irradiation performed with the sample partially immersed in water.
