# Supplementary material for: Photobiomodulation and Oxidative Stress: 980 nm Diode Laser Light Regulates Mitochondrial Activity and Reactive Oxygen Species Production
Source: Oxid Med Cell Longev. 2021 Mar 3;2021:6626286. doi: 10.1155/2021/6626286 (PMC7952159; doi:10.1155/2021/6626286)
Supplement: Supplementary Materials — Figure 1 supplementary: experimental design. Mitochondria were isolated from bovine liver. Mitochondria samples were irradiated at the room-air temperature or with the tube sample immersed in water. The samples were then processed for the biochemical analysis. Figure 2 supplementary: representations of sample temperature behaviour during the experiments. Before: temperature of the sample before irradiation. Room-air after: temperature of the sample after irradiation performed at room-air temperature. Room-air after+reagents: temperature of the sample after irradiation performed at a room-air temperature and the addition of reagents for biochemical evaluation. Water after: temperature of the sample after irradiation performed with the sample partially immersed in water. [file 6626286.f1.docx]

##
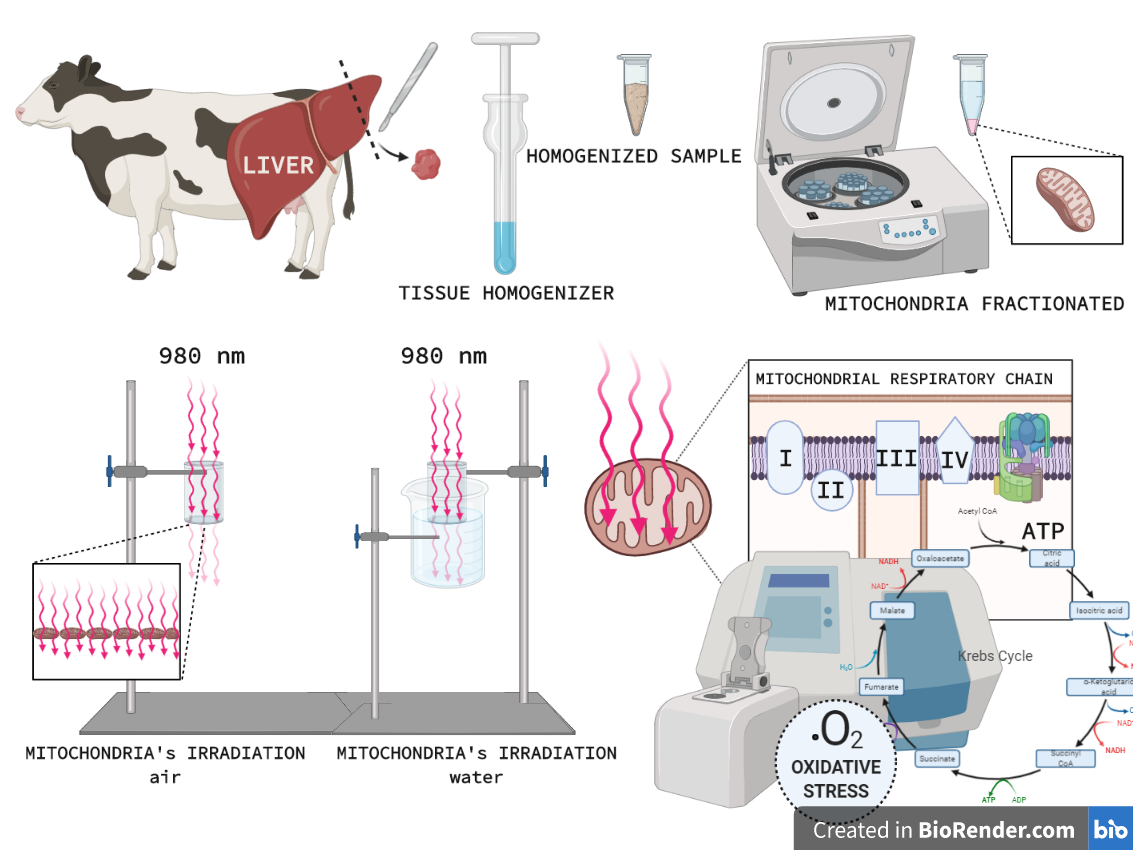
Supplementary Materials

**Figure 1 supplementary**: Experimental Design. Mitochondria were isolated from bovine liver. Mitochondria samples were irradiated at the room-airtemperature or with the tube sample immersed in water. The samples were then processed for the biochemical analysis.


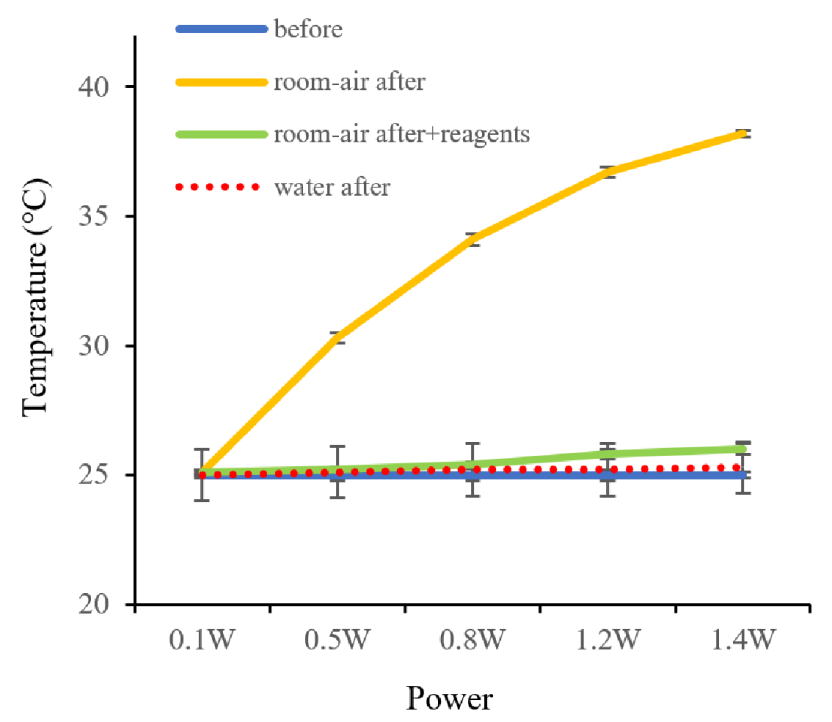


**Figure 2 supplementary**: Representations of samples temperature behaviour during the experiments. Before= temperature of the sample before irradiation. Room-air after= temperature of the sample after irradiation performed at room-air temperature. Room-air after+reagents= temperature of the sample after irradiation performed at room-air temperature and the addition of reagents for biochemical evaluation. Water after= temperature of the sample after irradiation performed with the sample partially immersed in water.
